# AF-6 Protects Against Dopaminergic Dysfunction and Mitochondrial Abnormalities in *Drosophila* Models of Parkinson’s Disease

**DOI:** 10.3389/fncel.2017.00241

**Published:** 2017-08-10

**Authors:** Adeline H. Basil, Joan P. L. Sim, Grace G. Y. Lim, Shuping Lin, Hui Ying Chan, Simone Engelender, Kah-Leong Lim

**Affiliations:** ^1^Neurodegeneration Research Laboratory, National Neuroscience Institute Singapore, Singapore; ^2^Department of Physiology, National University of Singapore Singapore, Singapore; ^3^National University of Singapore Graduate School for Integrative Sciences and Engineering Singapore, Singapore; ^4^Department of Biochemistry, Rappaport Faculty of Medicine and Research Institute, Technion-Israel Institute of Technology Haifa, Israel; ^5^Neuroscience and Behavioral Disorders Program, Duke-NUS Medical School Singapore, Singapore

**Keywords:** parkin, LRRK2, mitochondria, mitophagy, *Drosophila*, dopamine

## Abstract

Afadin 6 (AF-6) is an F-actin binding multidomain-containing scaffolding protein that is known for its function in cell-cell adhesion. Interestingly, besides this well documented role, we recently found that AF-6 is a Parkin-interacting protein that augments Parkin/PINK1-mediated mitophagy. Notably, mutations in Parkin and PINK1 are causative of recessively inherited forms of Parkinson’s disease (PD) and aberrant mitochondrial homeostasis is thought to underlie PD pathogenesis. Given the novel role of AF-6 in mitochondrial quality control (QC), we hypothesized that AF-6 overexpression may be beneficial to PD. Using the *Drosophila melanogaster* as a model system, we demonstrate in this study that transgenic overexpression of human AF-6 in parkin and also pink1 null flies rescues their mitochondrial pathology and associated locomotion deficit, which results in their improved survival over time. Similarly, AF-6 overexpression also ameliorates the pathological phenotypes in flies expressing the Leucine Rich Repeat Kinase 2 (LRRK2) G2019S mutant, a mutation that is associated with dominantly-inherited PD cases in humans. Conversely, when endogenous AF-6 expression is silenced, it aggravates the disease phenotypes of LRRK2 mutant flies. Aside from these genetic models, we also found that AF-6 overexpression is protective against the loss of dopaminergic neurons in flies treated with rotenone, a mitochondrial complex I inhibitor commonly used to generate animal models of PD. Taken together, our results demonstrate that AF-6 protects against dopaminergic dysfunction and mitochondrial abnormalities in multiple *Drosophila* models of PD, and suggest the therapeutic value of AF-6-related pathways in mitigating PD pathogenesis.

## Introduction

Parkinson’s disease (PD) is a prevalent neurodegenerative movement disorder that is characterized pathologically by the progressive loss of dopaminergic neurons in the substantia nigra pars compacta of the midbrain (Dorsey et al., 2007). This results in a severe depletion of striatal dopamine (DA) that is needed for an individual to execute proper, coordinated movements. Although most cases of PD occur in a sporadic manner, a subset of PD cases is inheritable and attributable to mutations in specific genes (Martin et al., 2011). Among these, recessive mutations in *Parkin* and dominant mutations in *leucine rich repeat kinase 2 (LRRK2)* are currently recognized to be the most prevalent cause of early-onset and late-onset familial PD respectively (Martin et al., 2011). The pivotal role that Parkin plays in maintaining dopaminergic neuronal survival is underscored by our current recognition that Parkin dysfunction represents not only a predominant cause of familial parkinsonism but also a formal risk factor for sporadic PD (Dawson and Dawson, 2010). Indeed, Parkin is widely accepted to act as a broad spectrum neuroprotectant (Zhang et al., 2015). Parkin normally functions as a ubiquitin ligase that targets substrates for ubiquitin modifications. A key advancement in our understanding of how parkin could afford neuroprotection came from recent revelation that Parkin functions as a crucial mediator of mitochondrial quality control (QC)—the optimal maintenance of which is especially important for neurons that are well known to be metabolically demanding. In this model, Parkin collaborates with another PD-linked gene product known as PINK1 to target damaged mitochondria for removal via a specialized form of autophagy known as “mitophagy” (Jin and Youle, 2012). Consistent with this, we and others have demonstrated that disease-associated Parkin mutations compromise its role in mitochondrial QC (Lee et al., 2010; Lim et al., 2012), thereby providing a mechanism underlying neuronal death in PD.

Over the years, several factors that participate in Parkin/PINK1-mediated mitophagy have been elucidated. Among these is Afadin 6 (AF-6), a F-actin binding, multidomain-containing scaffolding protein that is better known for its function in cell-cell adhesion. AF-6 partners with a wide variety of structural, junction and signaling molecules to form adherens junctions that facilitate cell-cell adhesion (Rikitake and Takai, 2011). Interestingly, we recently found that AF-6 interacts with Parkin and augments Parkin/PINK1 mitophagy pathway (Haskin et al., 2013). Importantly, we further found that AF-6 is present in Lewy bodies and that its levels are strikingly decreased in the striatum and substantia nigra of sporadic PD patients, suggesting that deficient AF-6 levels may contribute to the accumulation of dysfunctional mitochondria observed in the disease. Given these findings, we hypothesized that AF-6 overexpression may be beneficial to PD. Using the *Drosophila melanogaster* as an *in vivo* model system, we show here that transgenic overexpression of human AF-6 in parkin and also pink1 null flies rescues their mitochondrial pathology and associated locomotion deficit, which results in their improved survival over time. Similarly, AF-6 overexpression also ameliorates the pathological phenotypes in flies expressing the LRRK2 G2019S mutant. These include the restoration of mitochondrial size and DA level that are otherwise abnormal in the brains of LRRK2 G2019S mutant flies. Conversely, when endogenous AF-6 expression is silenced, it aggravates the disease phenotypes of LRRK2 mutant flies. Finally, we found that AF-6 overexpression is also protective against the loss of dopaminergic neurons in flies treated with rotenone, a mitochondrial complex I inhibitor commonly used to generate animal models of PD (Johnson and Bobrovskaya, 2015). Taken together, our results demonstrate that AF-6 protects against dopaminergic dysfunction and mitochondrial abnormalities in multiple *Drosophila* genetic models of PD, suggesting the therapeutic value of AF-6-related pathways in mitigating PD pathogenesis.

## Materials and Methods

### Fly Stocks

Fly lines for *Ddc*-Gal4 (expresses in dopaminergic and serotonergic neurons), *24B*-Gal4 (muscle-specific driver), *Elav*-Gal4 (pan neuronal driver), *yw*, *w*^1118^, UAS-LRRK2 G2019S, UAS-mito-GFP, parkin null flies (*park1*) and pink1 null flies (B9) were purchased from the Bloomington *Drosophila* Stock Center (Bloomington, IN, USA). UAS-AF-6 expressing flies lines were generated via microinjection of pUAST-AF-6 (derived from PRK5-HA-AF-6 via NotI/KpnI digestion) into fly embryos by BestGene Inc (Chino Hills, CA, USA). UAS-canoe RNAi was purchased from VDRC (Vienna *Drosophila* Stock Center, Austria).

### Climbing Assay, Flight Assay and Rotenone Treatment

Climbing assays were carried out according to previously described methods (Ng et al., 2009). Briefly, 20 female adult flies from each group were randomly selected after anesthetization and placed in a vertical plastic column (length 30 cm; diameter 1.5 cm). Age-matched normal flies were used as controls. After a 2-h recovery period from CO_2_ exposure, flies were gently tapped to the bottom of the column and the number of flies that reached the top of column at 1 min was counted. Results are presented as mean ± SEM of the scores obtained from three independent experiments. In rotenone-treated flies, flies were fed with cornmeal-agar medium containing 50 μM rotenone (purchased from Sigma Aldrich) immediately after eclosion and during the entire experimental period, as described previously (Ng et al., 2009). For the flight assay, a 30 cm-tall bottle was filled with water to a height of 2 cm and a funnel was placed at the bottle opening. Flies from a vial were expelled into the bottle via the funnel. The flies found on the walls after their explusion indicated their flight ability whilst the flies on the water indicated their inability to fly. The number of flies for both outcomes was recorded accordingly. Flies were then retrieved from the bottle via anesthetizing with CO_2_.

### Immunohistochemistry of Fly Brains

Immunohistochemical analysis of whole-mount adult fly brains were prepared according to published protocols (Whitworth et al., 2005). Brains were dissected from adult flies in phosphate buffered saline (PBS) and fixed in 0.1 M HEPES with 4% (w/v) paraformaldehyde overnight at 4°C. Subsequently, they were washed with 0.2% (v/v) Triton X-100 in PBS and blocked with 5% (v/v) normal goat serum (NGS) for 1 h at room temperature. This is followed by incubation with primary antibodies (1:300 rabbit anti-tyrosine hydroxylase, Pel-Freeze and 1:250 mouse anti-GFP, Sigma Aldrich) overnight at 4°C. Brain tissues were then washed with 0.2% Triton X-100 for 1 h and incubated with secondary antibodies (1:400 FITC-conjugated goat anti-mouse and 1:300 Rhodamine-conjugated goat anti-rabbit, Jackson ImmunoResearch) overnight at 4°C. The following day, specimens were washed with 0.2% Triton X-100 for 1 h and mounted with Vectashield onto glass cover-slips for confocal microscopy (Olympus). The quantification of the dopaminergic cluster and mitochondrial area (size of mito-GFP puncta expressed as mean ± SD with *n* ≥ 10 DA neurons per experimental group) using ImageJ software were as described previously (Ng et al., 2012).

### Transmission Electron Microscopy Analysis and Dopamine Assay via HPLC

For TEM analysis, indirect flight muscles were prepared according to previous protocol (Ng et al., 2012) and specimens were sent for commercial EM analysis at the EM Research Services, Newcastle University (UK). For DA measurement, 10 fly heads were homogenized in 0.5 N perchloric acid and the lysates were filtered through 0.1 μm Durapore Ultrafree Centrifugal filters (Milipore, USA) prior to HPLC measurement.

### Statistical Analysis

Unpaired Student’s *t*-test is used to calculate the statistical *p* value in comparison between groups (*p < 0.05, **p < 0.001).

## Results

### Transgenic Overexpression of Human AF-6 in *Drosophila* Does Not Trigger Overt Phenotypes

**Figure 1:**
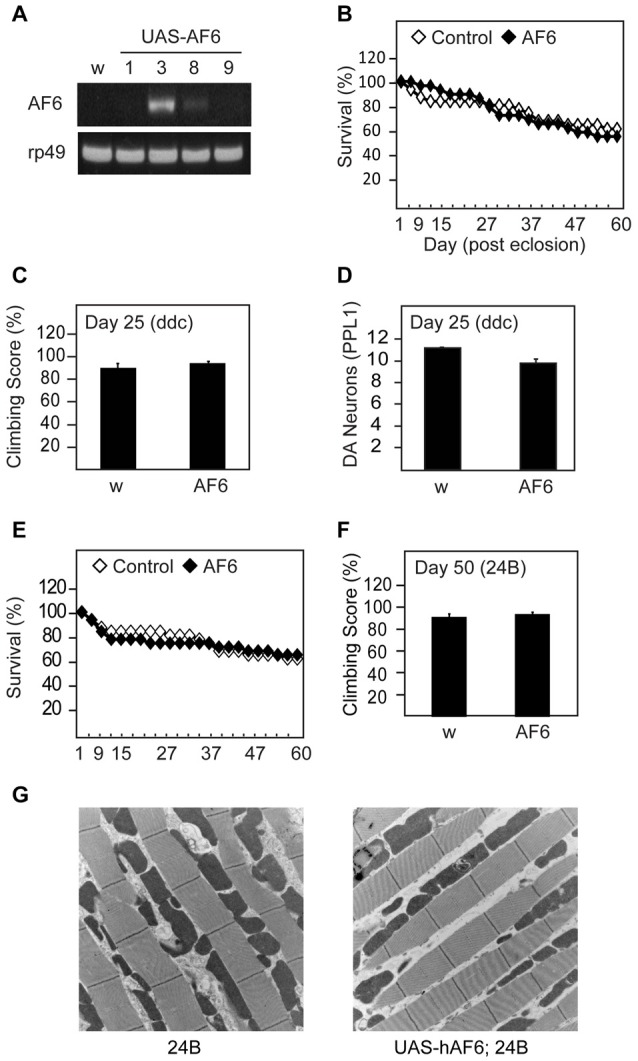
Transgenic overexpression of human Afadin 6 (AF-6) in *Drosophila* does not trigger overt phenotypes.** (A)** RT-PCR of four UAS-AF-6 transgenic fly lines (1, 3, 8 and 9) driven by *Elav* Gal4 driver. *White* flies (*w*^1118^) were used as a control. **(B)** Survival assay depicting the percentage of surviving AF-6 over-expressing flies with *Ddc* Gal4 driver (*UAS-AF-6*/+; *Ddc*/+) up to day 60 post eclosion. **(C)** Climbing score of control (*w*) and AF-6 transgenic flies at day 25 post eclosion. **(D)** Bar-graph showing the number of TH-positive dopamine (DA) neurons in the PPL1 cluster of control (*w*) and AF-6 transgenic flies at day 25 post eclosion. **(E)** Survival curve for AF-6 over-expressing flies with *24B* GAL4 driver (*UAS-AF-6*/+; *24B*/+) over 60 days. **(F)** Climbing score of control (*w*) and AF-6 transgenic flies at day 50 post eclosion. **(G)** TEM images of indirect flight muscles of control (*24B/+*) and (*UAS-AF-6*/+; *24B*/+) flies.

To examine the effects of AF-6 overexpression in flies, we generated *Drosophila* lines that express human AF-6 cDNA as a transgene under the regulation of the GAL4/UAS system (Figure 1A). We selected line #3 that clearly expresses human AF-6 at a robust level for the majority of our investigations. When driven by the *Ddc*-GAL4 driver (that restricts the expression of the transgene largely to TH-positive dopaminergic neurons and serotonergic neurons), AF-6 expression has negligible effects on the survival of adult flies up to 60 days post-eclosion (Figure 1B). Further, *Ddc*-GAL4 driven AF-6 expression in flies also exert no apparent effects on their climbing score (Figure 1C) or dopaminergic neuronal number (not shown), particularly in the PPL1 cluster that is known to be selectively susceptible to degeneration in several genetic PD fly models (Figure 1D). Similarly, when driven by the muscle-specific *24B*-GAL4 driver, AF-6 expression neither affects the survival nor locomotion ability of these flies (Figures 1E,F). Consistent with this, we did not detect prominent signs of mitochondrial abnormalities in the indirect flight muscles of *24B*-GAL4 driven AF-6 expressing flies relative to their control counterparts (Figure 1G). Taken together, our results demonstrate that AF-6 overexpression alone does not produce any overt phenotypes in *Drosophila*.

### AF-6 Overexpression Rescues the Disease Phenotypes of Parkin and Pink1 Null Flies

**Figure 2:**
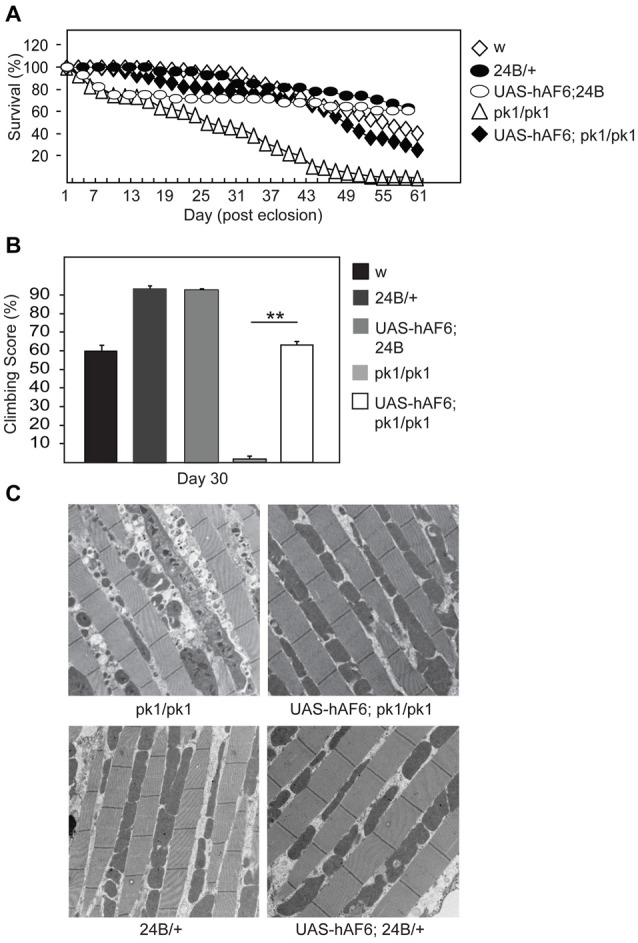
AF-6 overexpression rescues the disease phenotypes of parkin null flies.** (A)** Survival assay comparing the percentage of surviving flies between control (*w*^1118^), *24B/+, UAS-AF6/+* and parkin null (*pk1/pk1*) flies in the absence and presence of AF-6 overexpression driven by *24B* Gal4 driver (*UAS-AF-6*/+; *24B* > *pk1*) up to 60 days. **(B)** Climbing score of control and parkin null flies in the absence and presence of AF-6 overexpression in muscles using the *24B* driver at day 25 post eclosion. **(C)** TEM images of indirect flight muscles of control and parkin null flies in the absence and presence of AF-6 overexpression. **p < 0.001; unpaired student’s *t* test.

**Figure 3:**
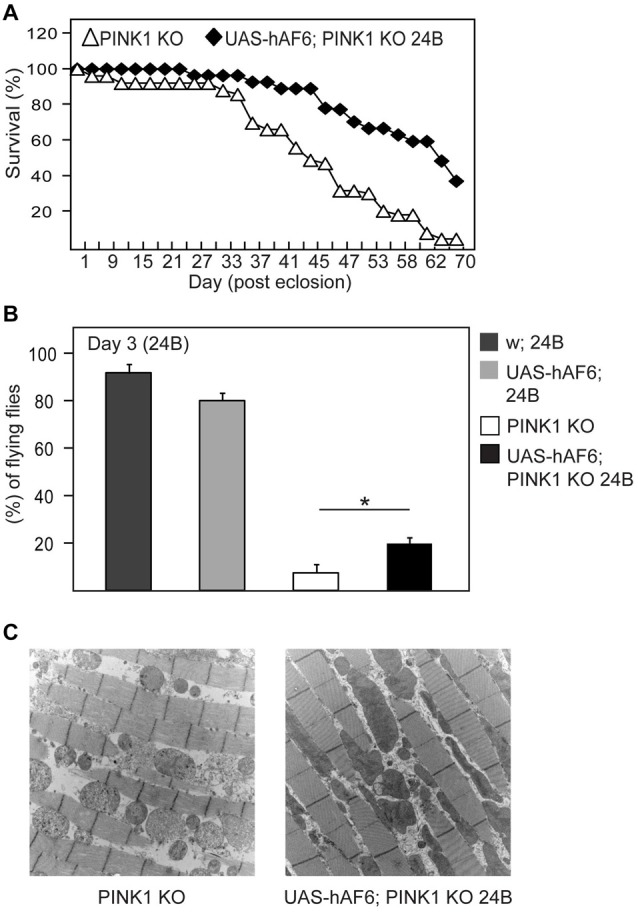
AF-6 overexpression rescues the disease phenotypes of pink1 null flies. **(A)** Survival curve depicting the percentage of surviving flies between pink1 knockout (*PINK1B9*) flies in the absence and presence of AF-6 overexpression with *24B* GAL4 driver (*UAS-AF-6*/+; *24B* > PINK1KO) up to 70 days. **(B)** Flight assay showing the percentage of pink1 knockout flies that are capable of flying in the absence and presence of AF-6 overexpression in muscles using the *24B* GAL4 driver at day 3 post eclosion. **(C)** TEM images of indirect flight muscles of pink1 knockout flies in the absence and presence of AF-6 overexpression. *p < 0.05; unpaired student’s *t* test.

Next, we examined the effects of AF-6 expression in parkin null flies. Notably, several groups including ours have reported that *Drosophila* parkin mutants display increased mortality and prominent mitochondrial pathology in their indirect flight muscles that is accompanied by marked locomotion defects (Greene et al., 2003; Wang et al., 2007). Interestingly, we found that AF-6 expression (via the *24B*-GAL4 driver) in parkin null flies dramatically enhances their survival (Figure 2A). Moreover, the double mutant flies also exhibit significantly improved climbing scores that is comparable to the performance by their control counterparts (Figure 2B). Remarkably, the muscle mitochondrial pathology associated with parkin null flies is virtually rescued in the presence of AF-6 overexpression (Figure 2C). Similar results were obtained when we repeated the experiment with another AF-6 overexpressing line (i.e., line #8; Supplementary Figure S1). Thus, AF-6 could apparently compensate for the loss of parkin function, the normal activity of which is needed for optimal mitochondrial QC. As Parkin and PINK1 functions are intimately interwoven, we also examined the effects AF-6 expression in pink1 null flies. Like *Drosophila* parkin null mutants, pink1-deficient flies are plagued with prominent mitochondria abnormalities that severely impair their flight ability and increase their mortality (Clark et al., 2006; Park et al., 2006). Again, we found that AF-6 overexpression (via the *24B*-GAL4 driver) enhances the survival and flight ability of pink1 null flies (Figures 3A,B) and markedly rescues their muscle mitochondrial pathology (Figure 3C).

### AF-6 Overexpression Rescues the Disease Phenotypes of LRRK2 Mutant Flies

**Figure 4:**
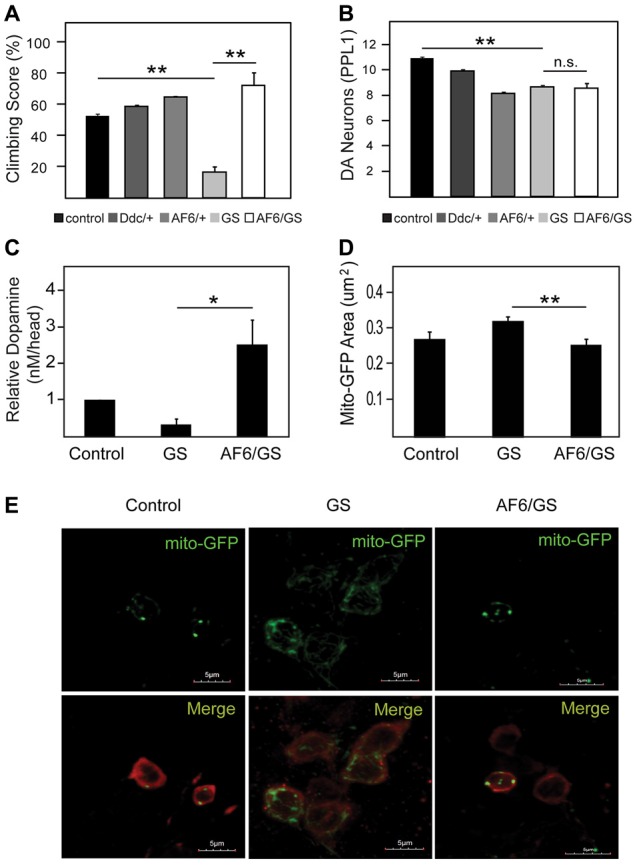
AF-6 overexpression rescues the disease phenotypes of leucine rich repeat kinase 2 (LRRK2) mutant flies. **(A)** Climbing score and **(B)** DA neuronal count (PPL1 cluster) of control (*yw*), *Ddc/+, UAS-AF-6/+* and LRRK2 G2019S (GS) mutant flies in the absence or presence of AF-6 overexpression using *Ddc* Gal4 driver at day 50 post eclosion. **(C)** Relative DA levels in these flies as measured by the HPLC. **(D)** Bar-graph showing the average size ± SEM of mito-GFP punctae in control (*yw*), GS mutant flies and GS/AF-6 double mutant flies at day 50 post eclosion. **(E)** Representative confocal microscopy images showing the localization of mito-GFP (green) in TH-positive neurons (red) of control (*yw*), GS mutant flies and GS/AF-6 double mutant flies. *p < 0.05, **p < 0.001; unpaired student’s *t* test.

We have previously demonstrated that LRRK2 G2019S expression in flies also result in mitochondrial abnormalities that leads to dopaminergic neuronal loss and climbing deficits, and that Parkin overexpression can ameliorate these pathological phenotypes in LRRK2 mutant flies (Ng et al., 2012). Given our demonstration above that AF-6 overexepression can rescue the mitochondrial pathology of parkin null flies, we sought to examine whether the same strategy might work in reducing the pathologies of LRRK2 mutant flies. For this purpose, we crossed AF-6 expressing flies with transgenic LRRK2 mutant flies to create a double transgenic *Drosophila* line that co-expresses AF-6 and LRRK2 G2019S. As previously reported (Ng et al., 2012), *Drosophila* expressing LRRK2 G2019S (via *Ddc*-GAL4) exhibit significant impairment in their climbing ability (Figure 4A). However, in the presence of AF-6 co-expression, we recorded a marked improvement in the climbing performance of LRRK2 mutant flies (Figure 4A). As the expression of AF-6 and LRRK2 G2019S is driven by the *Ddc*-GAL4 driver in this case, we also counted the dopaminergic neurons in the PPL-1 cluster that we and others have previously reported to be affected in LRRK2 mutant flies (Ng et al., 2012). Surprisingly, AF-6 overexpression does not appear to appreciably retard the loss of PPL-1 dopaminergic neurons in LRRK2 mutant flies (Figure 4B). However, HPLC analysis revealed that the DA level that is deficient in LRRK2 mutant flies is remarkably restored in the presence of AF-6 overexpression (Figure 4B), suggesting that AF-6 helps to improve the function of dopaminergic neurons in LRRK2 mutant flies that leads to their improved climbing performance (Figure 4C). Finally, we also examined the status of the neuronal mitochondria in LRRK2 mutant flies in the absence or presence of AF-6 co-expression via the mito-GFP assay. Consistent with our previous report, LRRK2 G2019S expression (via *Ddc*-GAL4) triggers the appearance of significantly enlarged mitochondria in TH-positive dopaminergic neurons (Figures 4D,E). Notably, this abnormality is significantly mitigated in the double transgenic AF-6/LRRK2 G2019S expressing flies. Taken together, our results demonstrated that AF-6 overexpression is an effective strategy in mitigating the pathologies of several *Drosophila* genetic models of PD that represent recessive and dominant parkinsonism.

### Silencing of AF-6 Expression in *Drosophila* LRRK2 Mutant Aggravates its Pathological Phenotype

**Figure 5:**
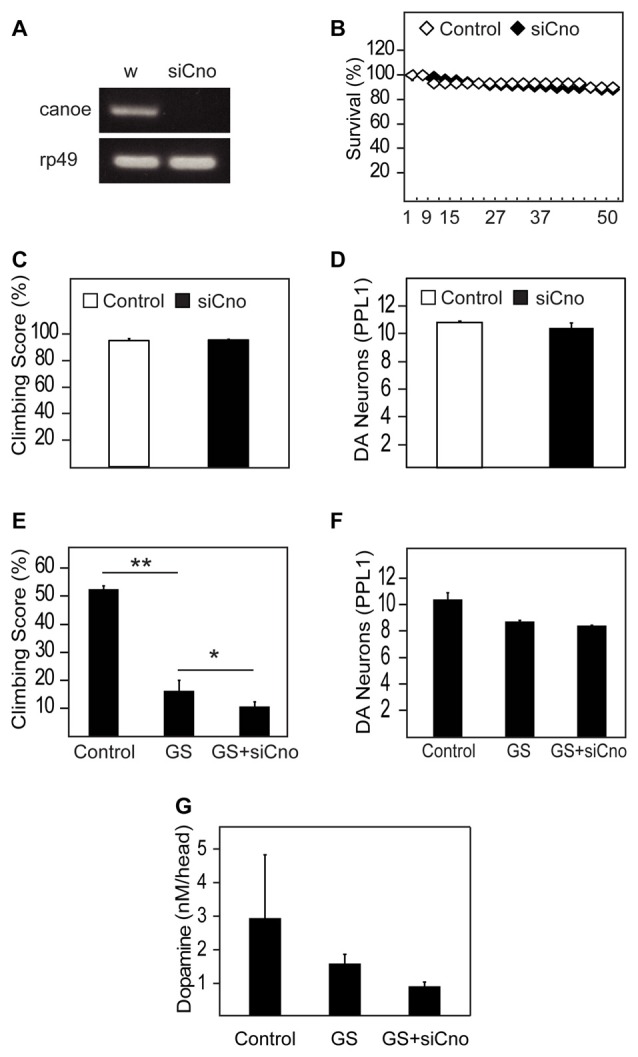
Silencing of AF-6 expression in *Drosophila* LRRK2 mutant aggravates its pathological phenotype.** (A)** RT-PCR showing the reduced expression of canoe (*cno*), the ortholog of AF-6, in flies relative to control flies (*w*^1118^). **(B)** Survival curve for canoe knockdown in DA neurons of flies with *Ddc* driver (*UAS-canoe* RNAi/+; *Ddc*/+) compared to control (*w)* flies up to day 50 post eclosion. **(C)** Climbing score and **(D)** DA neuronal count (PPL1 cluster) of control (*w*) and canoe knockdown flies with *Ddc* driver at 50 days post eclosion. **(E)** Climbing score and **(F)** DA neuronal count (PPL1 cluster) comparing between control (*yw*) and GS mutant flies in the absence and presence of canoe knockdown. **(G)** DA levels in these flies as measured by the HPLC system. *p < 0.05, **p < 0.001; unpaired student’s *t* test.

To examine whether AF-6 deficiency might promote LRRK2-induced neurotoxicity, we knocked down the expression of *canoe*, the orthologous counterpart of AF-6 in flies, via a siRNA strategy. When driven by the pan-neuronal *Elav*-GAL4 driver, the extent of silencing of canoe expression is remarkable (Figure 5A), which illustrates the efficiency of the siRNA-based strategy. When the knockdown of canoe expression is restricted to TH-positive neurons via the *Ddc*-GAL4 driver, we did not observe any obvious effects on the mortality, climbing performance or dopaminergic neuronal number of these flies relative to their control counterparts (Figures 5B–D), suggesting that canoe expression is not essential for the physiological functions of dopaminergic neurons under normal conditions. However, in the presence of LRRK2 G2019S expression, the silencing of *canoe* expression aggravates the locomotion deficits of the mutant flies (Figure 5E). Again, the dopaminergic neuronal count does not appear to be significantly different between LRRK2 G2019S flies and those with reduced canoe expression (Figure 5F), although the DA level in the latter group is evidently lower than LRRK2 mutant flies (Figure 5G), which might explain their poorer performance in climbing. We also carried out experiments to silence the expression of *canoe* via the *24B*-GAL4 driver, which is more relevant to our investigation on its effects on parkin and pink1 null flies as these mutant *Drosophila* exhibit prominent mitochondrial pathology in their muscles. However, *24B*-mediated silencing of* canoe* expression alone results in significantly accelerated mortality that precludes our intended line of investigation (Supplementary Figure S2). Indeed, nearly 70% of the 24B-si*canoe* flies died after a mere week post-eclosion (Supplementary Figure S2).

### AF-6 Overexpression Mitigates Rotenone-Induced Dopaminergic Neurotoxicity

**Figure 6:**
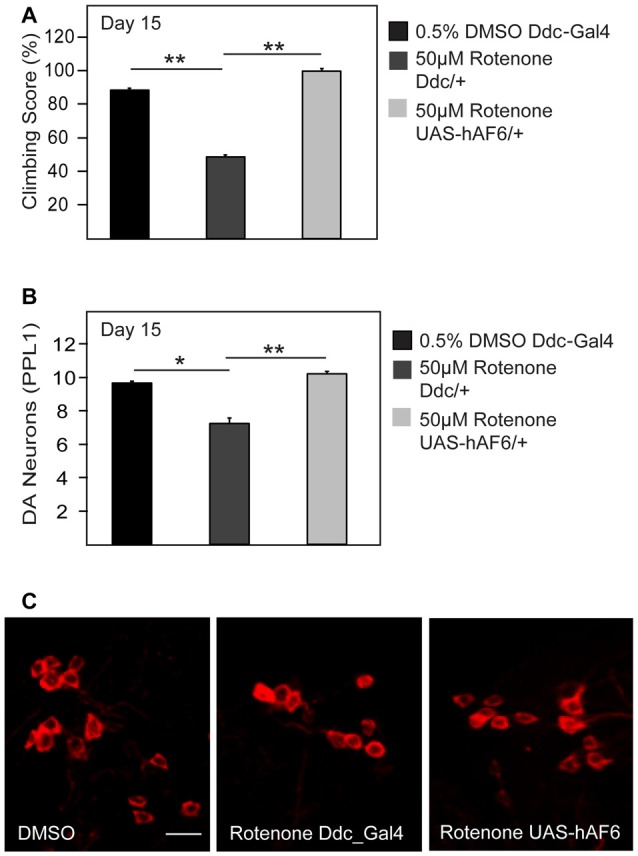
AF-6 overexpression mitigates the neurotoxicity of rotenone. **(A)** Climbing score and **(B)** Bar graph showing the number of TH-positive DA neurons (PPL1 cluster) of control *Ddc*/+ flies (treated with 0.5% DMSO), *Ddc*/+ and *UAS-AF-6*/+ after 15 days of exposure to 50 μM rotenone treatment using the *Ddc* Gal4 driver. **(C)** Representative confocal microscopy images showing TH-positive (red) DA neurons in the PPL1 cluster. *p < 0.05, **p < 0.001; unpaired student’s *t* test.

Given the apparent neuroprotective role of AF-6 overexpression in genetic fly models of PD that are associated with mitochondrial dysfunction, we wondered whether the strategy could also provide protection against exposure to PD-related mitochondrial toxins. To address this, we treated control and AF-6 transgenic flies with rotenone, a well established neurotoxin that induces PD via its inhibition of mitochondrial complex I (Johnson and Bobrovskaya, 2015). We and others have previously reported that rotenone-treated flies exhibit marked dopaminergic neurodegeneration and associated climbing defect (Coulom and Birman, 2004; Ng et al., 2009). Consistent with this, we found that control flies treated with rotenone display these parkinsonian phenotypes prominently (Figures 6A–C). In contrast, *Drosophila* overexpressing AF-6 (via the *Ddc*-GAL driver) that are treated with rotenone performed significantly better in their climbing score compared to their similarly treated control counterparts (Figure 6A). Indeed, they are as good a climber as untreated control flies. In good correlation with this, rotenone-mediated dopaminergic neuronal loss is significantly retarded in AF-6 overexpressing flies (Figures 6B,C). Thus, AF-6-mediated neuroprotection similarly works in a fly PD model induced by a toxin that is well known to inhibit mitochondrial function.

## Discussion

Taken together, we have demonstrated in this study that transgenic overexpression of human AF-6 in several genetic and a toxin-induced fly model of PD rescues their pathological phenotypes including dopaminergic dysfunction and associated locomotion deficit as well as mitochondrial abnormalities. Conversely, when endogenous AF-6 expression is silenced, it aggravates the disease phenotypes of the LRRK2 mutant flies that we have examined. Our results thus support the widely-held notion that mitochondrial dysfunction underlies the pathogenesis of PD and at the same time, suggest a neuroprotective role for AF-6 in PD.

At face value, AF-6 seems like an unlikely candidate to contribute to neuroprotection in PD. After all, the majority of studies to date focus on the role of AF-6 in cell-cell adhesion, the deficiency of which in mice results in embryonic lethality due to the disorganization of cell–cell junctions during embryogenesis (Zhadanov et al., 1999). However, when the ablation of AF-6 function is confined to neurons, perforation in hippocampal synapses occurs, suggesting that the protein participates in the formation and remodeling of synapses (Majima et al., 2009; Beaudoin et al., 2012). Consistent with this, AF-6 knockdown in cortical pyramidal neurons results in simplification of the dendritic fields and a concurrent loss of excitatory synapses, suggesting that AF-6 is important for the maintenance of dendritic structure and synaptic transmission (Srivastava et al., 2012). Whether AF-6 plays a role in the nigrostriatal dopaminergic pathway that is relevant to PD is relatively unexplored, although a previous report demonstrated that rasagiline-mediated rescue of dopaminergic neurons in MPTP-treated parkinsonian mice involves the activation of a spectrum of Trk-related signaling components including AF-6 (Mandel et al., 2007). More recently, we found that AF-6 could augment the Parkin/PINK1 pathway to promote the removal of damage mitochondria via mitophagy (Haskin et al., 2013), which suggests the possible involvement of AF-6 in PD. Although it is intriguing to note that a cell membrane-localized protein (i.e., AF-6) can participate in the mitophagy process, we found that the localization of AF-6 to the mitochondria may be promoted by its interaction with Parkin (Haskin et al., 2013). There is precedent for this dual residency of AF-6 as Buchert et al. (2007) have previously demonstrated that AF-6 has the ability to translocate to the nucleus to regulate cell growth processes. Thus, cellular AF-6 may be located in multiple compartments depending on the condition. This may also reflect the pleiotropic functions of AF-6 that is (as alluded above) not confined to the formation of cell junctions but also in cell migration, polarization, differentiation, proliferation and survival (Mandai et al., 2013). Moreover, at least two forms of AF-6 exist (I and S forms) and the protein can interact with a plethora of other protein partners including various transmembrane proteins and intracellular signaling molecules (Mandai et al., 2013). However, at this moment, we do not know how the differential compartmentalization of AF-6 is regulated or how the multiple functions of AF-6 may be linked together. Indeed, we cannot exclude the possibility that AF-6-mediated protection may involve its various extra-mitochondrial roles. Notwithstanding this, our previous report (Haskin et al., 2013) represents the first documented relationship between AF-6 and mitochondrial QC. Thus, we recognized that there is still a paucity of information regarding how AF-6 is able to regulate this mitochondrial-related process. Here, we have extended the relevance of our previous findings to the *in vivo* context that is related to the pathogenesis of PD. Importantly, our previous study also revealed that AF-6 is present in Lewy bodies, and that its levels are strikingly decreased in the striatum and substantia nigra of sporadic PD patients (Haskin et al., 2013), suggesting that decreased AF-6 levels may contribute to the disease. In a reciprocal manner, it is attractive to speculate that enhanced AF-6 expression may be beneficial to PD. This is precisely what we have observed in this study.

That AF-6 overexpression can rescue the phenotypes of parkin, pink1 and LRRK2 mutant flies that represent various recessive and dominant forms of PD that would position AF-6 as an important player in the pathogenesis of the disease. Moreover, the same strategy can also afford protection against rotenone-induced dopaminergic neurotoxicity in flies. However, the mechanism that underlies AF-6-mediated protection seen in the *Drosophila* PD models remains to be elucidated, although our previous (Haskin et al., 2013) and current results suggest that it likely exerts its neuroprotective properties through enhancing mitochondrial QC. Notwithstanding this, it is curious to note that AF-6 could preserve mitochondrial integrity in the absence of Parkin (i.e., in parkin null flies), as we have previously demonstrated that Parkin is essential for AF-6 augmentation of mitophagy (Haskin et al., 2013). Consistent with this, we found that AF-6 overexpression alone in the absence of Parkin could not rescue mitophagy defects in HeLa cells treated with CCCP (not shown). How AF-6 could apparently protect against mitochondrial dysfunction in the absence of Parkin (i.e., in parkin null flies) remains to be clarified. Interestingly, Zhang et al. (2011) found that AF-6 is a substrate of AMPK-mediated phosphorylation. This finding is of interest to us because we have recently found that AMPK activation mitigates mitochondrial pathology induced by parkin deficiency and protects against dopaminergic neurodegeneration in *Drosophila* models of PD (Ng et al., 2012). We further found that AMPK likely exerts its neuroprotective function via PGC-1α, a key regulator of mitochondrial biogenesis (Ng et al., 2017). Whether AF-6 could enhance PGC-1α-mediated mitochondrial biogenesis remains to be clarified. Nonetheless, these findings together with our results suggest the possibility of a parallel pathway involving AMPK-PGC-1α and AF-6 in the maintenance of mitochondrial homeostasis that is important for dopaminergic neuronal function and survival. Clearly, more work needs to be done but our current results sufficiently emphasize the potential value of understanding the role AF-6-related pathways in PD pathogenesis.

## Ethics Statement

Invertebrate animals, i.e., *Drosophila* were used that is exempted from ethics approval.

## Author Contributions

AHB, JPLS, GGYL, SL and HYC performed the experiments and analyzed the data. SE and K-LL conceived and organized the study. K-LL and AHB wrote the manuscript with contributions from SE.

## Conflict of Interest Statement

The authors declare that the research was conducted in the absence of any commercial or financial relationships that could be construed as a potential conflict of interest.
