# Supplementary material for: AF-6 Protects Against Dopaminergic Dysfunction and Mitochondrial Abnormalities in Drosophila Models of Parkinson’s Disease
Source: Front Cell Neurosci. 2017 Aug 10;11:241. doi: 10.3389/fncel.2017.00241 (PMC5554356; doi:10.3389/fncel.2017.00241)
Supplement: Supplementary file 1 [file Image_1.pdf]

Supplemental Information

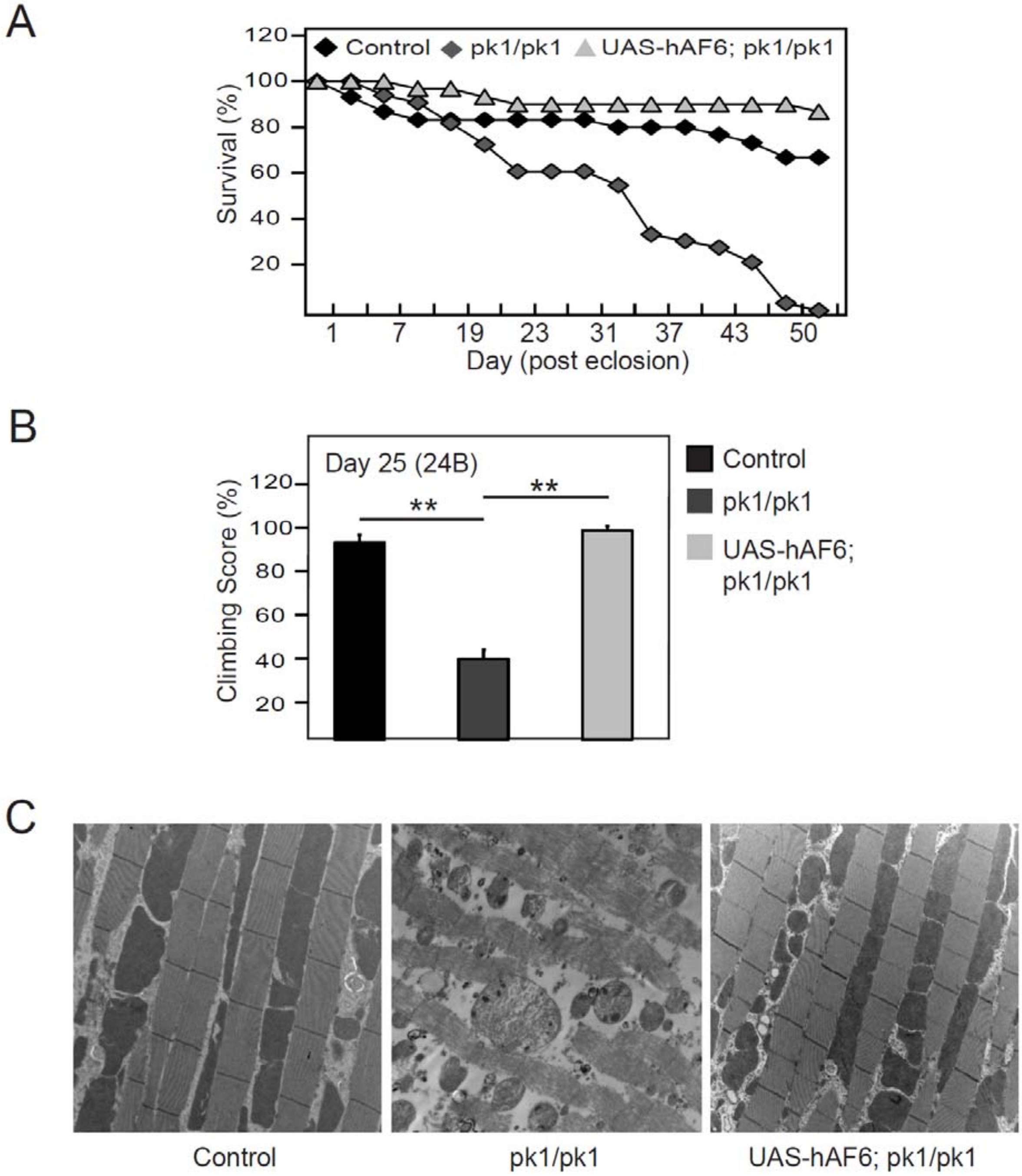

Figure S1. AF-6 overexpression rescues the disease phenotypes of parkin null flies. (A) Survival assay comparing the percentage of surviving flies between control (w1118) and parkin null (pk1/pk1) flies in the absence and presence of AF-6 (line 8) overexpression (UAS-AF6/+;24B>pk1) up to 50 days. (B) Climbing score of control and parkin null flies in the absence and presence of AF-6 (line 8) overexpression in muscles using 24B driver at day 25 post eclosion. (C) TEM images of indirect flight muscles of parkin null flies in the absence and presence of AF-6 (line 8) overexpression. **$p < 0.001$; unpaired student's *t* test.

Supplemental Information

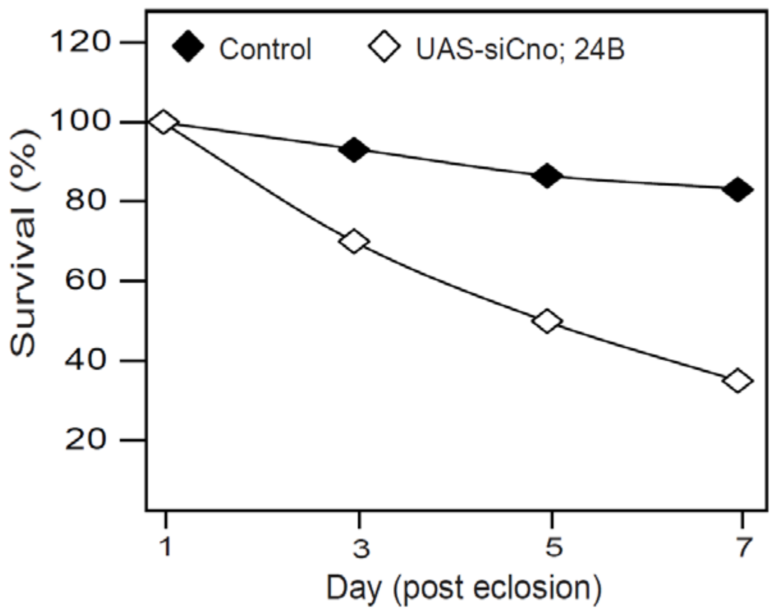

Figure S2. Muscle-specific silencing of canoe expression results in marked mortality. Survival assay comparing the percentage of surviving flies between control (w1118) and *24B*-driven sicanoe (siCno) flies.
